# A Critical Review on the Design, Manufacturing and Assessment of the Bone Scaffold for Large Bone Defects

**DOI:** 10.3389/fbioe.2021.753715

**Published:** 2021-10-14

**Authors:** Yi Huo, Yongtao Lu, Lingfei Meng, Jiongyi Wu, Tingxiang Gong, Jia’ao Zou, Sergei Bosiakov, Liangliang Cheng

**Affiliations:** ^1^ Department of Engineering Mechanics, Dalian University of Technology, Dalian, China; ^2^ DUT-BSU Joint Institute, Dalian University of Technology, Dalian, China; ^3^ Faculty of Mechanics and Mathematics, Belarus State University, Minsk, Belarus; ^4^ Department of Orthopeadics, Affiliated Zhongshan Hospital of Dalian University, Dalian, China

**Keywords:** bone scaffold, microstructure design, metamaterial, optimization, additive manufacture, performance assessment

## Abstract

In recent years, bone tissue engineering has emerged as a promising solution for large bone defects. Additionally, the emergence and development of the smart metamaterial, the advanced optimization algorithm, the advanced manufacturing technique, etc. have largely changed the way how the bone scaffold is designed, manufactured and assessed. Therefore, the aim of the present study was to give an up-to-date review on the design, manufacturing and assessment of the bone scaffold for large bone defects. The following parts are thoroughly reviewed: 1) the design of the microstructure of the bone scaffold, 2) the application of the metamaterial in the design of bone scaffold, 3) the optimization of the microstructure of the bone scaffold, 4) the advanced manufacturing of the bone scaffold, 5) the techniques for assessing the performance of bone scaffolds.

## Introduction

Every year, many surgeries of bone replacements are performed worldwide (Wang G. et al., 2018). In fact, the number of such bone surgeries keeps increasing as the aging of the population. The autograft and the allograft are the two main approaches for fixing the large bone defects in clinic. However, both of them have different shortcomings: The former has the disadvantages of donor site morbidity, the lack of bone supply, the nerve lesion, etc. The latter has genetic differences, the anatomic variations, the disease transmission, etc. Therefore, presently there is still no feasible solution for fixing the large bone defects.

**FIGURE 1:**
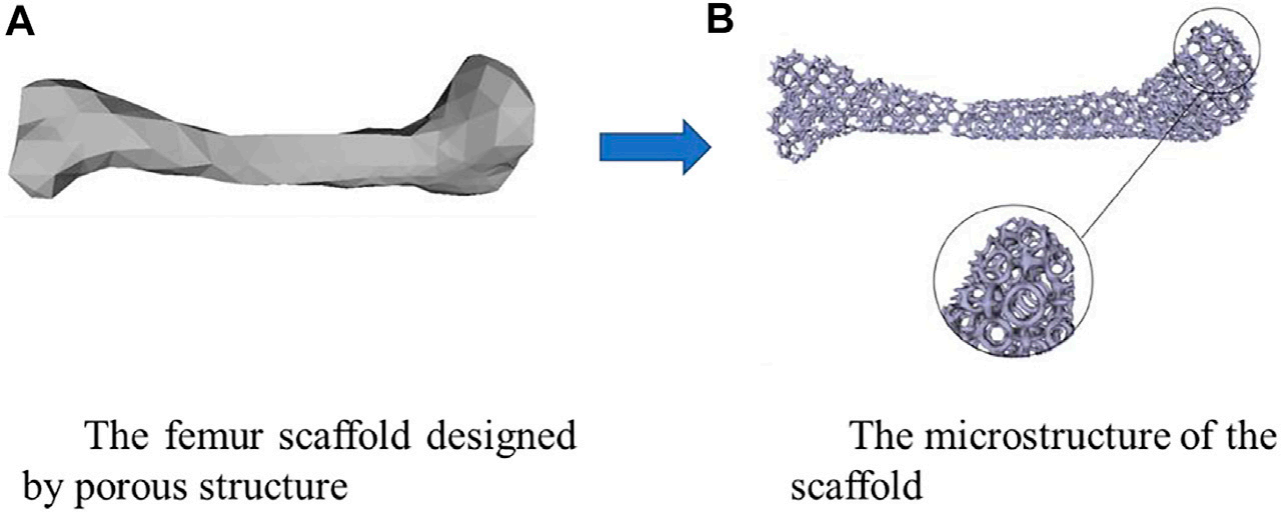
An example of designing the scaffold using the porous microstructure (adapted from Guo et al., 2018).

The bone scaffold is a promising method for fixing the bone defects and is currently under intensive investigations. However, there are still many issues to be solved urgently in the current bone scaffolds, the stress shielding and the interfacial loosing problems in the metal scaffolds and the early failure in the degradable scaffolds. Structural design is one of the crucial and effective approaches for solving these challenging issues. By using the structural design, some superior properties can be achieved, such as the superconductivity, the invisibility in the fields of aerospace and aviation (Wong et al., 2017). It should be noted that human bones and joints, including jaws and femurs, are not completely solid. Therefore, the bone scaffolds or replacements designed are porous and the design of the microstructure of bone scaffolds is more crucial than the design of the exterior shape of the scaffolds or replacements. The interconnected porous structure not only facilitates the inflow of nutrients and the treatment of metabolic waste, but also provides good conditions for cell growth and attachment. Additionally, the stress shielding can be avoided to a certain extent when the porous structures are used instead of the solid structures (Guo et al., 2018). An example of designing the femur scaffold using the porous microstructures is shown in Figure 1. Therefore, the porous structure has important research significance in the field of designing and manufacturing bone scaffolds. On the other hand, the biological properties are also very important. Autograft has been widely used to restore extensive or complex fracture, stem cells are widely used in tissue engineering approaches, and among these adult mesenchymal stem cells (MSCs) hold a great promise for regenerative medicine strategies. Furthermore, a correct stem cell source selection is crucial to achieve the bone treatment (Mattioli-Belmonte et al., 2015). The additive manufacturing (AM) which gives more freedoms for the structural design is an emerging technique for producing the structure with complex internal microstructures. However, how to make the appropriate structural design considering the AM process is still a challenging issue.

Regarding the design of bone scaffolds, it has been revealed in previous studies that the microstructure of the bone scaffold has a significant influence not only on its mechanical performance, but also on the cell behaviors, the degradation rate of the scaffold, etc. Therefore, structural design is a crucial step for advancing and expanding the performances of the scaffolds, and for solving the key problems in bone scaffolds. For example, the interfacial loosing issue may be solved by introducing the metamaterial into the bone scaffold, because the scaffolds with negative Poisson’s ratios will not compress the surrounding tissues under the compression scenario. On the other hand, the additive manufacturing (AM) is the emerging technique for producing the structures with complex internal microstructures and gives more freedoms for the structural design. However, how to make the appropriate structural designs considering the AM process is still a challenging issue.

The aim of the present study was to provide a critical review on the microstructural design, the advanced manufacturing and the performance evaluation of the bone scaffold and thus to forward the development of the bone scaffold in the relevant fields.

## Review on the Design of the Microstructure of the Bone Scaffold

The property of the scaffold highly depends on the microstructure of the scaffold. Therefore, the microstructure design of the scaffold is a crucial step in the design of bine scaffolds. In the past a few years, the microstructures of the bone scaffolds have emerged from the regular shapes to the irregular shapes, and from the periodic to the non-periodic structures. The microstructures of the scaffolds in the literature can be classified into three main groups (Table 1). First, the scaffolds are formed by the periodic regular lattices, e.g., the cube. This type of scaffold is mostly used in the early development of the bone scaffold and seldom investigated in the recent researches except for the investigations on some special properties of the scaffold, e.g., the cell attachment behavior. Second, the scaffolds are formed by the bionic microstructures, such as the triply periodic minimal surface (TPMS) based structures. The TPMS structures have been widely used in the design of bone scaffold due to their superior behaviors, such as a large surface-to-volume ratio, a mean curvature of zero, etc. The TPMS based scaffolds are still one of the main research topics nowadays. Third, the scaffolds are formed by the irregular and non-periodic structures (Wang H. et al., 2018). Because the irregular and non-periodic microstructures are also present in the natural porous human bones, this kind of scaffold has a large similarity to the natural bones and may be an ideal replacement for the defected bone tissues. However, some advanced mathematical algorithms (e.g., the Voronoi algorithm) are required to design this kind of scaffold, which is challenging and hinders its development.

**TABLE 1:** Three main classes of the microstructure of the bone scaffolds.

Time period	Representative microstructure		Advantages and disadvantages	References
Early stage	Regular periodic unit		Advantages: Easy for the design and optimization	Gómez et al. (2016)
	Cubes	Hexagons		
	Spheres	Cylinders	Disadvantages: Poor mechanical and flow properties	
Approximately 2012 to present	TPMS based unit		Advantages: Large surface-to-volume ratio, fully connected, diverse types and controllability	Al-Ketan and Abu (2019)
	Diamond	Gyroid		
	Schwarz P	Fischer-Koch S	Disadvantages: The clinical needs still cannot be fully met and there is room for improvement	
Approximately 2016 to present	Nonperiodic, irregular unit		Advantages: The complex and anisotropic microstructures of bone tissues can be simulated	Wang et al. (2018a)

			Disadvantages: The design and optimization are challenging	

In the first two types of scaffolds, the scaffolds are formed by unit cells and different methods can be used to form the scaffolds, among which the functionally graded, the hybrid, etc. have been widely used. In the functionally graded scaffolds, the porosity of the unit cell can be varied linearly, quadratically or exponentially in one or several directions (Liu et al., 2018). Additionally, the basic forming topology can be graded in one dimension of the scaffold (Al-Ketan and Abu, 2019). The hybrid forming method combines two basic topologies together to form a new structure. For example, Chen et al. (2019) have generated a hybrid TPMS structure using the hybrid method. Because of the combination, the advantages of the two basic topologies can be both maintained in the new structures. For example, the Neovius cellular structure possesses a high Young’s modulus but a small shear modulus, while the Schwarz P cellular structure possesses a low Young’s modulus but a high shear modulus. Then the hybrid structure based on these two structures possesses both a high Young’s modulus and a high shear modulus (Chen et al., 2019). A summary of different microstructures of the bone scaffold is shown in Table 2.

**TABLE 2:** Some commonly used approaches for designing different microstructures of bone scaffolds.

Formation method	Representative microstructure	Advantages and disadvantages	References
Linearly functionally graded scaffold	Relative density grading	Advantages: The overall performance of the structure can be easily optimized	Al-Ketan et al. (2020)
	Cell size grading		
Exponentially functionally graded scaffold	One structure	Disadvantages: Some special properties cannot be achieved and there is still room for improvement	Al-Ketan and Abu (2019)
	Different structure		
Hybrid scaffold		Advantages: The advantages of various structures can be combined	Chen et al. (2019)
		Disadvantages: The design and optimization are challenging	

Over the past a few years, the microstructure of the bone scaffold has emerged from simple regular shape to irregular, bionic structures, which has significantly improved the mechanical and biological properties of the bone scaffolds. However, it should be pointed out that there is still a large room to improve the design of scaffolds. For example, the application of the smart structures (e.g., structures with negative Poisson’s ratio, negative thermal coefficient) into the design of the bone scaffold has been seldom explored.

## Review on the Application of the Metamaterial in the Bone Scaffold

In recent years, the metamaterials have drawn the attentions of many researchers, because some special properties can be achieved using the metamaterials, such as the super-toughness, invisibility, etc. The metamaterials are artificially engineered structures not found in the nature and constituent materials (Singh and Marwaha, 2015). The metamaterials can be divided into the auxetic and non-auxetic structures, the difference between which is that the auxetic structure has the negative Poisson’s ratio. In the present study, their applications in the field of porous bone replacement are reviewed.

First, regarding the auxetic structures, there are mainly three different types: the re-entrant, the chiral and the rotating structures. The re-entrant structures can be formed by the arrowhead structure, the star shape structure, the missing rib structure, etc. (Hou and Silberschmidt, 2015). Under the tensile loading, the re-entrant structure exhibits the negative Poisson’s ratio (NPR) effect. The structures with the re-entrant unit possess some superior properties, such as the exceptional fatigue performance. Some researchers have applied the auxetic structures in the design of bone replacements and superior performance has been found. For example, Kolken et al. (2021) showed that the re-entrant structure can restore the bone-implant contact in the lateral side of a hip stem.

**FIGURE 2:**
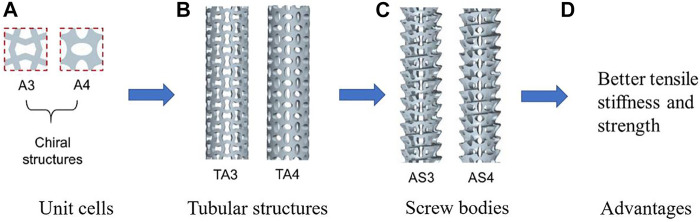
An example of using the metamaterial to design the porous bone screw (adapted from Yao et al, 2020).

The chiral structures are the ones with the deformation dominated by the rotational reflection and exhibit the NPR effect. The chiral structures have also been applied in the design of bone replacements. For example, (Yao et al, 2020), designed different structures including the re-entrant structures, the chiral structures and the rotating structures. Different bone screws were generated using these structures and the mechanical properties and fixation strength were evaluated. The results showed that the auxetic bone screws composed of re-entrant structures and chiral structures possess higher tensile stiffness and strength, and those composed of re-entrant structures and rotating structures possess better auxetic performance. An example of using the metamaterials to design the bone screws is shown in Figure 2.

The rotating auxetic structures are typically composed of the rigid squares connected through the simple hinges at their vertices. When a tensile loading is applied, the squares rotate at the vertices and the whole structure is expanded and the Poisson’s ratio equals to −1. The rotating auxetic structures can also base on the rigid congruent rectangular, the rigid equilateral triangles, the rigid rhombi and the rigid parallelograms. Regarding the application of this auxetic structure, it is shown in Yao et al.’s study (2020) that the bone screws composed of re-entrant structures and rotating structures possess better auxetic performances compared to the traditional structures.

**FIGURE 3:**
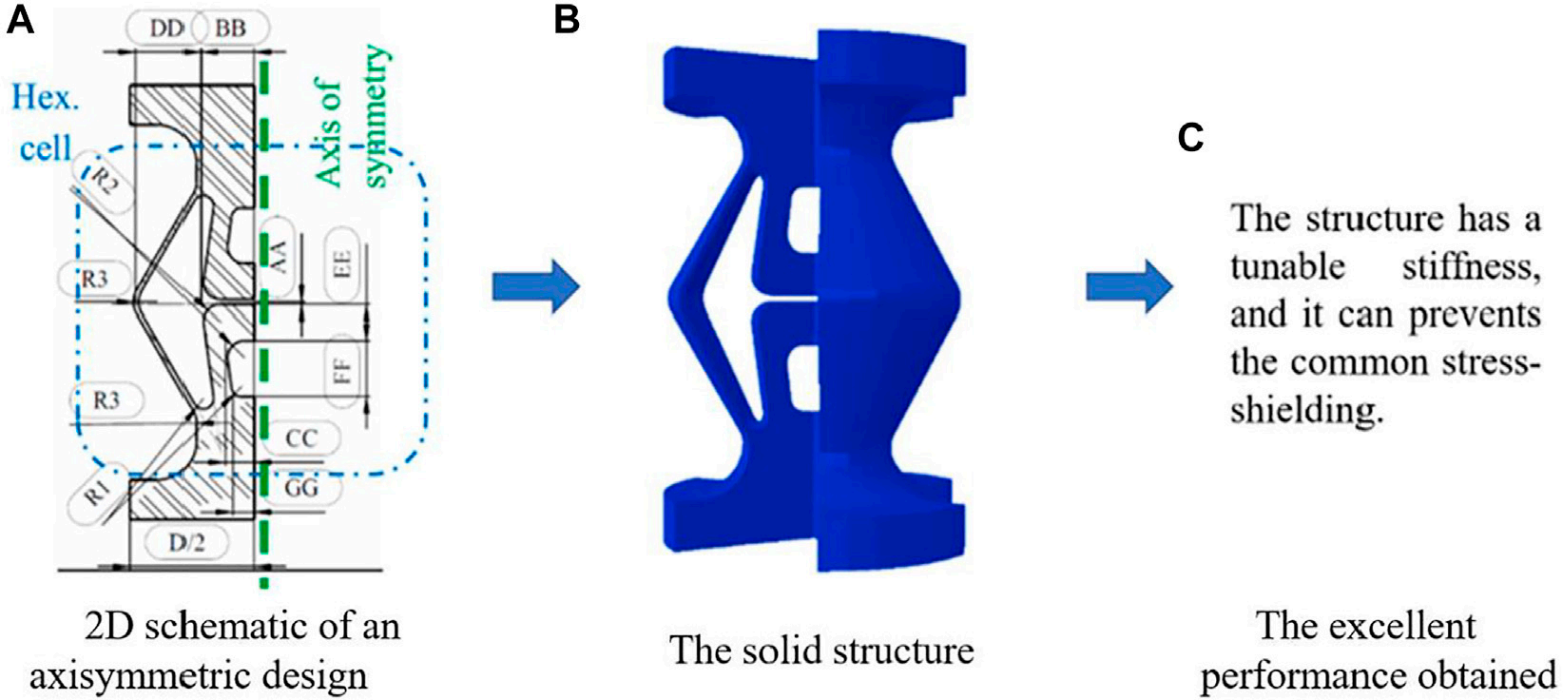
An example using the smart structure to design the bone replacement (adapted from Hashemi et al, 2021).

Besides the structures mentioned above, some new smart structures have also been designed and investigated in the literature. For example, Hashemi et al. (2021) designed a new structure that can be treated as auxetic structure to solve the problems such as the stress shielding and bone nonunion in the healing of fractured bones (Figure 3). The structures designed possess a tunable stiffness, and the results showed that the novel bone rods allow for the broken bones to move in a controlled fashion along the longitudinal axis. The motion stimulates the bone healing while it prevents the common stress-shielding of ordinary bone rods leading to osteoporosis.

In addition to the auxetic structures, the non-auxetic metamaterial can also be used to design the porous bone replacements. For example, the honeycomb structure is a type of non-auxetic metamaterial and has been used in the relevant fields. The honeycomb structure has the geometry of a honeycomb to allow for the minimization of the amount of used materials to reach the minimal. The geometries of the honeycomb structures can vary widely but the common feature of all such structures is an array of hollow cells formed between thin vertical walls. The cells are often columnar and hexagonal in shape (Wahl et al., 2012). With regard to the application of the honeycomb structure in the bone replacement, Wang et al. (2021) designed four groups of different honeycomb structures for the repair of human bone defects. The results showed that the honeycomb structures can meet the requirements of the static properties such as the elastic modulus, the yield strength, the permeability and the wall shear stress (Garcia Garcia et al., 2018). designed the hydroxyapatite 3D honeycomb structure for bone reconstruction. The results showed that the honeycomb structures are biomimetic supports promoting *in vitro* osteo-compatibility, osteo-conduction and osteo-induction and are suitable for the application of bone reconstruction in complex situations such as the repair of maxillofacial defects. Liu et al. (2018) fabricated the honeycomb microstructure for bone tissue engineering and found that the structure promotes cells adhesion, migration, proliferation and osteogenic differentiation on the scaffolds. A summary of the metamaterials and their applications in bone replacements is shown in Table 3.

**TABLE 3:** Summary of the metamaterials and their applications in bone implant.

Type of metamaterial		Application	Advantages and disadvantages	References
Auxetic structures	Re-entrant	Medical screw, bone-implant contact	Better tensile stiffness, good NPR effect, longer fatigue life	Kolken et al. (2021), Yao et al (2020)
	Chiral	Medical screw, bone scaffold fabrication	Better tensile stiffness, high fracture toughness, limited by chirality	Yao et al (2020)
	Rotating	Medical screw, auxetic materials fabrication	Better auxetic performance, low stability	Yao et al,(2020)
Non-auxetic	honeycomb	Bone defects repair, bone reconstruction, bone tissue engineering materials	Proper elastic modulus, high yield strength, high permeability	Garcia Garcia et al. (2018), Liu et al. (2020), Wang et al. (2021)

In summary, because some special properties can be achieved using the metamaterials, the application of the metamaterials is of great significance. However, the design and manufacturing of such structures may be challenging, which needs further intensive investigations in the future.

## Review on the Optimization of the Microstructure of the Bone Scaffold

Structural optimization is crucial for improving the performance of the bone scaffold. The objective function, the design variables and the constraints are the three key elements in establishing the optimization framework. Regarding the establishment of the objective function, three different types are widely used. First, one of the properties of the scaffold, such as the stiffness, permeability, is set as the objective property. The advantage of this method is that the specific objective property can be reached. However, when the scaffold is implanted into the human body, the sole property optimized cannot meet the multiple demands. Therefore, this method may not be the best. The second approach is to set up the objective function that based on the mechano-biological behaviors of the scaffold. For example, Xiao et al. (2018) optimized the scaffold to maximize the formation of the new bone tissues. Using this method, the interaction between the scaffold and the surrounding tissues can be considered, so as to prolong the life expectancy of the scaffold. However, considering only one property in the optimization process may not meet the multiple requirements. The third approach is to use the morphological and the effective properties of the human bone tissues to set up the objective function. Because the human bones are the optimized structure from thousand years’ evolution, the scaffolds with the morphological and mechanical properties similar to those of the natural bones may be the best and the performance of this type of scaffold may be very good. Using this approach, Wieding et al. (2014) have established the optimization framework for bone scaffold. However, it should be noted that in the previous studies, the properties of the bone and the scaffolds are considered only at one time point. When the scaffolds are implanted into the human body, it will be working for many years during which period the surrounding environment is changing. In the optimization of the bone scaffold, no previous study has considered the dynamic behaviors of the bone tissues and scaffolds. Regarding the setting up of the optimization variables, the dimensions of the scaffolds are set as the design variables for the regular scaffolds. For the TPMS scaffold, the constants appeared in the TPMS mathematical equations are set as the design variables (Chen et al., 2019). A summary of the optimization of the microstructure of bone scaffolds in shown in Table 4.

**TABLE 4:** State-of-art review on the optimization of the microstructure of bone scaffolds.

Type of microstructure	Objective function	Design variables	References
Scaffold with the dimension explicitly expressed	Maximize the scaffold stiffness or permeability	Stiffness	Xiao et al. (2018)
		Permeability	Dias et al. (2014)
	Maximize the newly formed bones	Computational mechanobiological model	Boccaccio et al. (2018)
		Geometrical parameters	Montazerian et al. (2019)
	Minimize the difference between the designed scaffold and the human bone	Biomechanical stability, Young’s modulus	Wieding et al. (2014)
Scaffold with the dimension implicitly expressed	Maximize the scaffold stiffness or permeability, or the minimum of the flexibility	Stiffness	Hollister and Lin (2007)
		Volume fraction	Xiao et al. (2012)
	Minimize the difference in the morphology, mechanical properties between the scaffold and the human bone	Geometrical parameters	Shi et al. (2018)
		Porosity, Young’s modulus and pore size	Vijayavenkataraman et al. (2018)
Non-periodic, irregular bionic microstructure	Minimize the difference in the morphology, mechanical properties between the scaffold and the human bone	Histomorphometry indices of trabecular bone	Gomez et al. (2016)
		Porosity	Wang et al. (2018b)

As for the constraints in the design and optimization of the scaffolds, many constraints have to be considered. These constraints can be divided into three main categories: the biological constraints, the mechanical constraints and the constraints associated with the additive manufacturing. First, regarding the biological constraints, when designing the bone scaffolds for replacing the large bone defects, at least the following biological constraints should be considered: 1) the microstructure of the scaffold should be inter-connected to allow for the free flow of the fluid, 2) the porosity of the scaffold should be larger than 50% to facilitate the bone ingrowth and 3) the pore size should be between 50.0 and 800.0 µm to ensure the bone ingrowth. Second, regarding the mechanical constraints and requirements, at least the following constraints should be considered: 1) the mechanical properties of the scaffold should match those of the surrounding tissues, so that the stress shielding can be reduced or eliminated, 2) the mechanical properties of the scaffold should meet the anatomic loading requirements to avoid the mechanical failure of the scaffold. Third, regarding the AM constraints, the constraints associated with the SLM should at least include the followings: 1) the minimal thickness of the scaffold structure should be larger than 0.2 mm, 2) the minimal hanging angle should be larger than a threshold value, which depends on the specific manufacturing technique (Zhang et al., 2019). It should be noted that the manufacturing constraints will be changed if different AM techniques are used. A summary of the constraints required to be considered in the design of the bone scaffolds is shown in Table 5.

**TABLE 5:** The constraints required to be considered in the design of the bone scaffolds.

Constraint type	The constraint	References
Biological constraint	Connectivity of the microstructure	Heinl et al. (2008)
	Porosity of the scaffold no less than 50%	
	pore size should be larger than 0.05 mm and less than 0.8 mm	
Mechanical constraint	Close to the modulus of the surrounding tissue	Wang et al. (2016)
	The anatomic loading requirements should be met	
Additive manufacturing constraint	Structure thickness no less than 0.2 mm if the SLM is used	Arabnejad et al. (2016)
	The minimal hanging angle should be larger than a set threshold value	Zhang et al. (2019)

In summary, the optimization of the microstructure of the bone scaffold is of great significance, because the mechanical properties of the bone scaffolds depend on their microstructures. However, the design of scaffold is challenging, partially due to the difficulties in setting up the objective function and the constraints required to be considered in the design. In the present, there is still no consensus in the objective function and some constraints are still difficult to be considered. Consequently, the porous structures designed still cannot meet the clinical needs and requirements. Therefore, there is still room for improvement in the microstructure of bone scaffold, which needs further research.

## Review on the Manufacturing of the Bone Scaffold

**FIGURE 4:**
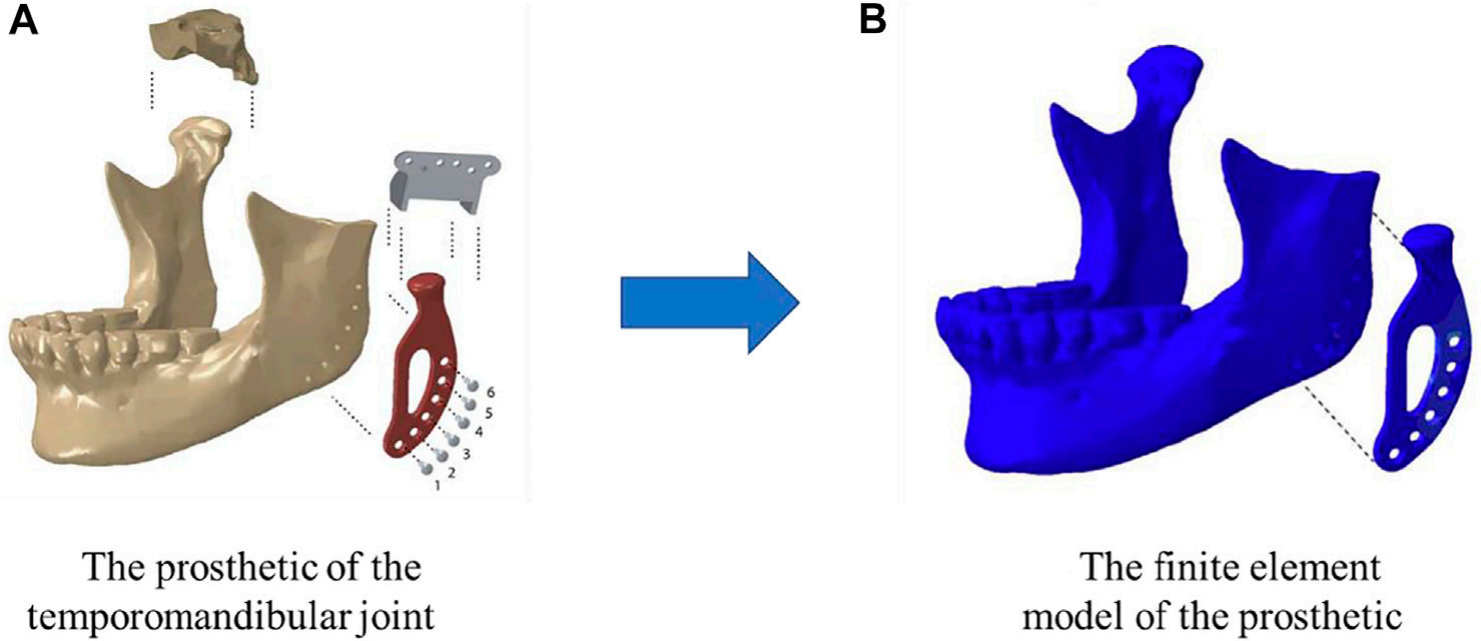
A temporomandibular joint produced by 3D print (adapted from Ackland et al., 2017).

After designing and optimizing the microstructure of the scaffolds, the next step is to manufacture the designed structures. Because of the complicated internal structures of the scaffolds and for the purpose of personalized treatment, the AM (3D) technique is widely used to produce the scaffolds. The AM-printed bone scaffolds are also widely used in practice. For example, Ackland et al. (2017) fabricated a personalized 3D-printed prosthesis for a patient requiring total joint replacement surgery of the temporomandibular joint (Figure 4). The AM is an emerging technique, which enables the production of the nonhomogeneous and irregular structures. Among the various AM techniques, the selective laser sintering (SLS), the selective laser melting (SLM), the electron beam melting (EBM) and the binder jetting (BJ) have been successfully used to produce the porous bone implants (Table 6). Below a brief introduction of these techniques and the application of these techniques in producing the porous bone scaffolds are given.

**TABLE 6:** The commonly used manufacturing methods for producing the scaffolds.

Manufacturing method	Advantages and disadvantages	References
Selective laser sintering	Advantages: Easy to incorporate multiple materials, no support structure	Madrid et al., 2019, Williams et al. (2005)
	Disadvantages: Relatively low densification and poor mechanical properties	
Selective laser melting	Advantages: Completely melt the powder, no support structure	Weißmann et al. (2016)
	Disadvantages: high power consumed, long processing time	
Electron beam melting	Advantages: Be able to process metals with an extreme high melting point	Ataee et al. (2018)
	Disadvantages: Limited to handling conductive metal materials	
Binder jetting	Advantages: High efficiency, no residual stress	Vangapally et al. (2017)
	Disadvantages: Low mechanical properties	

The SLS is to use the laser beam to scan the powders in the powder bed according to the path specified by the computer, and then bond and solidify the raw powder material on the working table. Since the structure to be built can be self-supporting by unmelted powder, no additional support is required, which is an obvious feature of SLS. However, some drawbacks are associated with the SLS. For example, the surface of the finished product is relatively rough. The products normally exhibit a poor formation quality due to the partial melting of the particles, and additionally the products possess relatively low densification and poor mechanical properties, which restricts their applications in the load bearing orthopedic scenarios (Madrid et al., 2019). Williams et al. (2005) fabricated the bone scaffolds using the SLS technology and the compressive modulus and yield strength are ranged from 52.0 to 67.0 MPa and from 2.0 to 3.2 MPa, respectively, which are comparable to those of human trabecular bones.

The SLM is another widely used AM technology, in which the metal powder is heated to complete melting and formed in the layer-by-layer fashion until the entire product is formed. Unlike the SLS, the SLM has higher laser energy and the powders can be completely melted during the processing without the binder (Dogon et al., 2020). Similar to the SLS, no support structure is required for the SLM technique. However, the SLM technique consumes a large amount of energy and takes a long time. Also, some partially melted particles are present on the surface of the product which causes the product produced by SLM has a high surface roughness. Using the SLM technique, some biomedical implants have been successfully produced. For example, (Weißmann et al., 2016 **)**, have fabricated the Ti6AL4V lattice structures and the results showed that the structures are strong enough to bear the impacting loads and an elastic modulus comparable to that of human cortical bone can be achieved.

The EBM is an AM technique using an electron gun to generate an electron beam to melt the metal powders. Compared with the SLS and SLM techniques, the primary advantage of EBM is its high beam-material coupling efficiency, which makes it easy when processing metals with an extreme high melting point (Hao and Harris, 2008). Therefore, it has been utilized to produce the porous metal scaffolds. For example, Ataee et al. (2018) produced Ti-6Al-4V Gyroid scaffolds using EBM and the produced exhibit high porosities ranging from 82 to 85%. In addition, the yield strength and elastic modulus obtained are in the range from 13.1 to 15.0 MPa and from 637.0 to 1084.0 MPa, respectively, which are comparable to those of trabecular bone.

The BJ is the AM technology using the powder and liquid binding agent (Gao et al., 2018). In the first step of this technique, the powder is bound through adhesion and chemical reaction. In the second step, the binder is removed, followed by the post-treatment process such as sintering, infiltration with a second material and HIP for densification. The advantage of the BJ is that it is cost-effective with a high efficiency and no residual stress is involved. However, shrinkage may occur during the sintering, and the mechanical properties of the product are poor and low. Generally speaking, the products produced using the BJ are basically particles glued together resulting in fragile structures with a limited mechanical performance. Using the BJ, Vangapally et al. (2017) have fabricated different lattice structures and the results showed that a high strength can be achieved in the AM printed bone scaffolds.

Another noteworthy aspect is the materials used to manufacture the bone scaffolds. Different materials will have an important impact on the mechanical and biological properties of the bone scaffolds. For example, biomedical cements mainly composed of the calcium phosphate compositions (CPC) have been widely applied for bone repair and regeneration (Kang et al., 2018). One of the promising injectable materials for bone repair and regeneration is the biomedical cement. Kang et al. (2018) reported a kind of cement which is made from mesoporous bioactive glass nanoparticles. The nanocement uses nano-sized powders while CPC uses micron-sized powders. Therefore, they will show different mechanical and biological properties. The Si ion release is a unique feature of the nanocement which contributes to the stimulation of cellular events, particularly angiogenesis. Compared with the traditional materials CPC, the nanocement shows some unique properties, which makes it a promising material for bone repair and regeneration. Singh et al. (2014) produced the magnetic nanofibrous scaffolds of poly and analyzed the mechanical and biological properties to find the efficacy for bone regeneration purpose. The results showed that the magnetic nanofibrous scaffolds have excellent cellular interactions. Similarly, Kim et al. (2014) produced the magnetic scaffolds and implanted them in rats. After 2 weeks, there were substantial fibroblastic cell and almost no inflammatory response. Besides, the strength and the elastic modulus are suitable for the bone regeneration.

## Review on the Evaluation of the Performance of the Bone Scaffold

**FIGURE 5:**
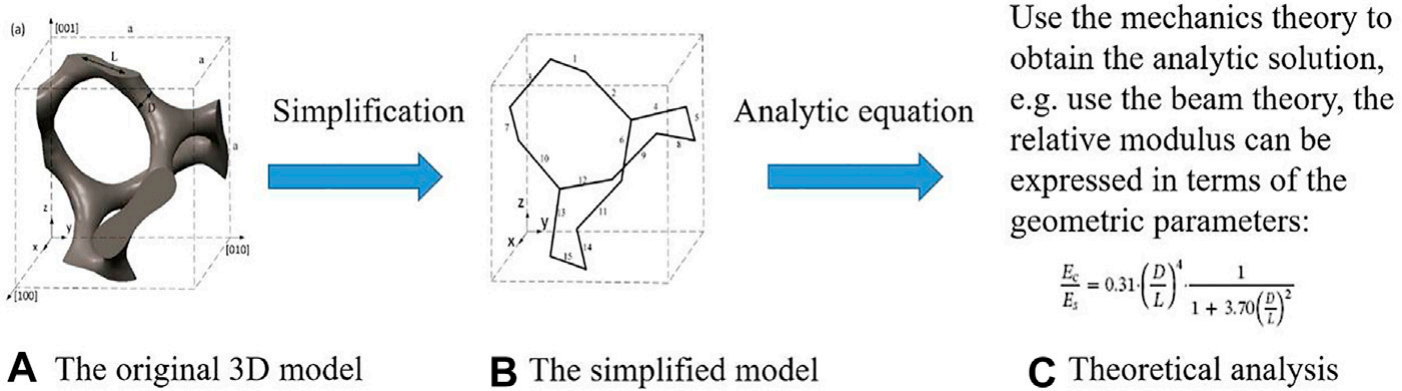
The analysis procedure for obtaining the properties of Gyroid structure using the analytical method (adapted from Yang et al., 2019).

The performance of the bone scaffold can be evaluated mainly using three types of methods: the in silico, the *in vitro* and the *in vivo* testing approaches. Regarding the in silico method, the analytical and numerical analysis can be used. The analytical method is to use the theories in the structural mechanics, the mechanics of materials, etc. to derive the formulas for the properties to be investigated. An example of this is shown in Figure 5, where the 3D Gyroid structure was first simplified into simple beam structures and then the beam theory was used to work out the relative elastic modulus of the structures (Yang et al., 2019 **)**. It is obvious that the simplification is the most crucial step in the analytical method. The model should be simplified to the extent that can be analytically solved but not oversimplified. Using the analytical method (Ahmadi et al, 2014), analyzed the mechanical properties of the cellular structures made of the diamond lattice unit cells. Using octahedral unit cells, Hedayati et al. (2017) analyzed the relationships between the mechanical properties of porous structures and their porosities, and furthermore the differences between the analytical and numerical results for elastic modulus and Poisson’s ratio were analyzed. Khodaei et al. (2018) analyzed the influence of scaffold porosity on the differences between the analytical and numerical results. It should be noted that although the analytical method can be used to quickly estimate some properties of the porous structures, the accuracy of it is low and the method should be mainly used for the qualitative analysis, e.g., the trend between the elastic modulus and the volume fraction of the Gyroid lattice (Yang et al., 2019).

**FIGURE 6:**
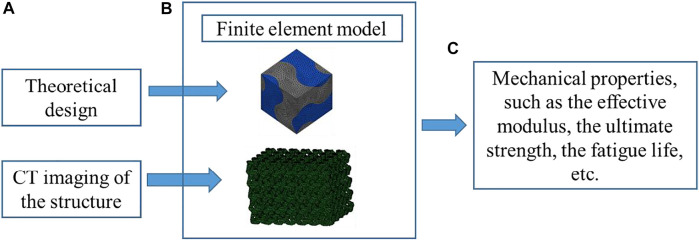
The analysis procedure for obtaining the properties of porous scaffolds using the finite element method (adapted from Lu et al., 2020a; Lu et al., 2020b).

Since the analytical method can only deal with the porous structures with the simple geometry and many assumptions and simplifications have to be made in the analysis, the numerical analysis method is more widely used than the analytical method. Compared with the analytical method, a higher accuracy can be obtained and the influence of structural dimensions on the results can be taking into account using the numerical method. Among the various numerical methods, the finite element (FE) modeling is the most widely used approach. The procedure for using the FE method to obtain the mechanical properties of porous scaffolds is shown in Figure 6. The FE models can be generated either from the theoretical design or the CT imaging of the structure, where the influence of the imperfect geometry can be taken into account in the latter approach. From the FE analysis, many mechanical properties of the porous structures can be obtained, such as the effective elastic modulus, the ultimate strength, the fatigue life, etc. Askari et al. (2020) analyzed the compressive mechanical properties using the scaffolds designed by the FE method. Adachi et al. (2006) simulated the formation of new bones and scaffold degradations using the FE approach. It should be noted that among various mechanical properties, the effective elastic modulus is the most reliable one predicted from the FE analysis (Lu et al., 2020b). The accurate prediction of the nonlinear mechanical properties, such as the ultimate strength, the fatigue life, relies on the accurate definitions of the material models. Because some uncertainties are still present in the nonlinear material models, simulating the nonlinear behaviors of scaffolds and bones is still challenging.

The advantages of the in silico method are that the property of the scaffold can be cost-effectively evaluated and the problem of the imprecise observations caused by the long time-consuming experiment can be avoided. However, it should be noted that some properties of the scaffold can be hardly evaluated using the in silico method, such as the fatigue, the cell behavior, etc. Even for the simple properties, such as the effective modulus, an experimental validation is normally required to ensure the numerical analysis can produce the valid results. Furthermore, it should be noted that many ideal situations are assumed in the in silico analysis. For example, the mechanical property of the base material is always assumed to be homogeneous and isotropic. Therefore, it is undoubtable that there are some discrepancies between the in silico analysis and the results from the experimental testing (Lu et al., 2020a). Nevertheless, the in silico analysis can still provide valuable results on the behaviors of different bone scaffolds (Lu et al., 2020b).

Regarding the *in vitro* testing method, various testing techniques can be used, such as the mechanical testing, the biological testing, etc. (Table 7). The mechanical testing is used to evaluate the mechanical properties of the porous structures. In the mechanical testing, different types of tests can be used, such as the uniaxial compression test, the three-point bending test, etc. So as to obtain the mechanical properties, such as the effective compressive modulus, the bending modulus, etc. For example, Castro et al. (2019) carried out the uniaxial compression test on Gyroid scaffolds for bone tissue engineering and the elastic moduli were obtained. In addition to the mechanical testing, the biological properties of the porous structures can also be assessed through the biological testing. For example, the permeability test can be used to evaluate the permeability of the porous structure (Dias et al., 2012; Montazerian et al., 2019). Meanwhile, the permeability can also be used to analyze the deformation of natural bone during the fluid flow (Syahrom et al., 2013). The degradation test can be used to assess the degradation rate of the porous structure (Wei et al., 2017; Yan et al., 2018). Last but not the least, cell culture experiments can be used to assess the biocompatibility (Xu and Simon, 2005), the osteoblast growth (Pon-on et al., 2014), the cell vitality and proliferation (Riboldi et al., 2005), cell compatibility of bone scaffolds (Li et al., 2016), etc. Furthermore, the differentiation of bone precursor cells is another aspect that can be reviewed. Mattioli-Belmonte et al. (2015) investigated the effect of scaffold structure on cell differentiation. The results showed that compared with the periosteum derived precursor cells (PDPCs), the periodontal ligament stem cells (PDL-SCs) are less osteoblastic committed. This may be caused by the scaffold structure. Mattei et al. (2015) introduced an experimental approach to investigate the contribution of substrate stiffness and other material-related cues in the modulation of stem cell fate. The results showed that both stiffness and hydroxyapatite content contribute to modulate osteoblastic-related gene expression in PDPCs. Chen et al. (2015) manufactured three-dimensional gradual porous polylactic-co-glycolic acid (PLGA) composite scaffolds and investigated the comparative and interactive effects of dynamic compression and SRY-related high-mobility group box gene-9 (SOX-9) on chondrogenesis of rabbit adipose-derived stem cells in the scaffolds. They showed that the scaffolds may benefit articular cartilage tissue engineering in cartilage regeneration for better force distribution. Dinescu et al. (2015) built a gelatin- (G-) alginate- (A-) polyacrylamide (PAA) 3D interpenetrating network (IPN), which is capable of supporting hADSCs proliferation and survival. It should be noted that the advantages of *in vitro* testing are that the specific property can be physically measured and compared to the in silico method, the physical features, such as the AM induced geometric defects in the scaffold, can be taken into account in the *in vitro* testing (Lu et al., 2020a). On the other hand, some properties, which can be hardly obtained using the numerical simulation such as the cell proliferation, can be obtained from the *in vitro* testing. However, it should be noted that in the *in vitro* testing, the scaffold is placed in the *in vitro* environment, which is different from that in the *in vivo* scenarios and because of this discrepancy, some *in vitro* testing results, such as those obtained from the cell culture testing, should be carefully interpreted.

**TABLE 7:** Summary of the *in vitro* testing methods.

Type of property	Testing methods	Properties to be evaluated	References
Mechanical properties	Compression test	Young’s modulus, compressive strength	Castro et al. (2019)
	Tensile test	Young’s modulus	Yan et al. (2018)
	Three-point bending test	Compressive strength	Yan et al. (2018), Xu and Simon, (2005)
	Fatigue test	Fatigue resistance	Bobbert et al. (2017)
Biological properties	Permeability test	Permeability of the scaffold	Dias et al. (2012), Montazerian et al. (2019)
	Degradation test	Degradation rate	Wei et al. (2017)
	Cell culture test	Cell availability, cell proliferation rate	Pon-on, et al. (2014)
	Immersion test	Weight change (degradation rate)	Yan et al. (2018)
	Viscosity tests	Fluid flow behavior	Wei et al. (2017)

Because the scenario created in the *in vitro* testing may be different from that in the *in vivo* scenarios, the *in vivo* testing as a crucial step before the clinical application is widely used to evaluate the behaviors of the porous scaffolds in the living environment (Table 8). Among the *in vivo* testing methods, cost-effective and time-efficiency, the animal testing is one of the crucial methods. Regarding the animal testing, it is important to choose the proper type of animal and sample from the animals. The criterion is that the animal sample should properly reflect the *in vivo* environment of the human body. Regarding the animal testing of the porous structure, different animals have been used in the literature. For example, Woodard et al., (2007) implanted the porous scaffold into the latissimus dorsi muscle of Yorkshire pigs and found that the implanted scaffolds exhibited a stress–strain response similar to that of cancellous bone with strengths between those of cancellous and cortical bones. Li et al., (2016) implanted the porous scaffold into the goat metatarsus and analyzed the stability of the implantation. It should be noted that the small animals, such as the mouse, are seldom used in the animal testing of bone scaffolds, because the mouse bone is too small to implant a porous scaffold. On the contrary, it is much easier to implant the scaffolds in the large animals, such as the goat, but some issues are associated with the experiments using the large animals. For example, the daily care of the large animals is challenging. Furthermore, due to the slow growth rate of the large animals, the testing period is normally much longer than that using the small animals. The human trial is the last test step before the large-scale clinical applications. Compared to the animal testing, the human trial takes much longer period and the variances between the human subjects may affect the performance of the porous scaffolds. Some human trials have been performed in the previous studies (Zeng et al., 2018). For example, Calori et al. (2008) compared the efficacy of recombinant bone morphogenetic protein 7 (rhBMP-7) and platelet-rich plasma (PRP) (both in collagen scaffolds) in the treatment of persistent fracture non-unions in 120 cases. Jager et al. (2009) treated ten patients with volumetric bone deficiencies in a study that used porous collagen I as a scaffold with MSCs and bone marrow aspirate in a 3-years follow-up. Sotome et al. (2016) assessed the efficacy and safety of the HAp/collagen scaffolds and found that it had the highest grade of bone regeneration but is associated with higher incidence of adverse effects.

**TABLE 8:** Summary of the *in vivo* testing methods.

Testing methods	Testing subject and site	Properties to be evaluated	References
Animal testing	Femur of the dog	Cell attachment and morphology	Lee et al. (2009)
	Goat metatarsus	Implant stability	Li et al. (2016)
	Dorsi muscle of Yorkshire pigs	Strength of the implanted scaffolds	Woodard et al. (2007)
	Skull bone of the New Zealand White rabbits	Combined bone and scaffold volume	Simon et al. (2007)
Human trial	Patella, proximal phalanx of the thumb, ulna	Bone regeneration	Sotome et al. (2016)

At present, there are only few reports on the *in vivo* testing of the scaffolds using human trial, because the clinical trials are a long process. The human trial is the crucial step for testing the scaffold in practice, and human clinical trials will be of great significance in the future.

## Conclusion and Future Perspectives

In the present article, the state-of-art progress in the design, manufacturing and assessment of the bone scaffold for fixing the large bone defects are reviewed. In conclusion, the microstructures of the bone scaffolds have evolved from the periodic regular unit to the non-periodic irregular unit, which can be designed using some advanced optimization algorithms. The additive manufacturing technique has enabled the production of the scaffolds with complicated internal structures. Various techniques can be used to assess the performance of the scaffold, especially the emerging cell culture experiments. However, there are still many issues remained to be solved, and to tackle these issues, further improvements and developments are still needed in the future, especially in the following perspectives:

1) To establish the optimization framework considering the dynamic interaction between the scaffold and surrounding tissues, especially the time-varying properties of both the degradable scaffolds and the bone tissues. In this challenge, the machine learning algorithm can be utilized to efficiently and quickly predict the dynamic behaviors of both the scaffolds and the surrounding tissues.
2) To improve the quality of the scaffolds produced by the additive manufacturing. This challenge can be tackled first by improving the AM technique, second by improving the design of the scaffold, e.g., incorporating the AM defects, etc. into the design stage to minimize the defects occurred in the products.
3) To comprehensively develop and apply the most advanced measurement techniques into the assessment of the performance of the scaffold. In recent years, the flexible measurement devices and many flexible and wireless sensors have been developed. These tools have enabled the fast and accurate measurements of some parameters (e.g., the surface strain, the muscle activation level) in the human body which can be hardly measured previously. Therefore, the performance of the scaffolds implanted in the human body can be better assessed using these up-to-date measurement techniques in the future.
